# A Maternal High-Energy Diet Promotes Intestinal Development and Intrauterine Growth of Offspring

**DOI:** 10.3390/nu8050258

**Published:** 2016-05-05

**Authors:** Peilin Liu, Long Che, Zhenguo Yang, Bin Feng, Lianqiang Che, Shengyu Xu, Yan Lin, Zhengfeng Fang, Jian Li, De Wu

**Affiliations:** 1 Key Laboratory of Animal Disease-Resistance Nutrition and Feed Science, Ministry of Agriculture, Sichuan Agricultural University, 211 Huimin Road, Wenjiang District, Chengdu 611130, Sichuan, China; sow_nutrition@sina.com (P.L.); chelong1989@126.com (L.C.); guoguo00002@163.com (Z.Y.); fengb123d@163.com (B.F.); clianqiang@hotmail.com (L.C.); shengyu_x@hotmail.com (S.X.); able588@163.com (Y.L.); fangzhenfeng@hotmail.com (Z.F.); lijian522@hotmail.com (J.L.); 2 Institute of Animal Nutrition, Sichuan Agricultural University, 211 Huimin Road, Wenjiang District, Chengdu 611130, Sichuan, China

**Keywords:** high energy diet, fetal growth, intestinal weight, insulin-like growth factor 1 receptor (IGF-1R)

## Abstract

It has been suggested that maternal nutrition during gestation is involved in an offspring’s intestinal development. The aim of this study was therefore to evaluate the effects of maternal energy on the growth and small intestine development of offspring. After mating, twenty gilts (Large White (LW) breeding, body weight (BW) at 135.54 ± 0.66 kg) were randomly allocated to two dietary treatments: a control diet (CON) group and a high-energy diet (HED) group, respectively. The nutrient levels of the CON were referred to meet the nutrient recommendations by the National Research Council (NRC, 2012), while the HED was designed by adding an amount of soybean oil that was 4.6% of the total diet weight to the CON. The dietary treatments were introduced from day 1 of gestation to farrowing. At day 90 of gestation, day 1 post-birth, and day 28 post-birth, the weights of fetuses and piglets, intestinal morphology, enzyme activities, and gene and protein expressions of intestinal growth factors were determined. The results indicated that the maternal HED markedly increased the BW, small intestinal weight, and villus height of fetuses and piglets. Moreover, the activities of lactase in fetal intestine, sucrase in piglet intestine were markedly increased by the maternal HED. In addition, the maternal HED tended to increase the protein expression of insulin-like growth factor 1 receptor (IGF-1R) in fetal intestine, associated with significantly increased the gene expression of *IGF-1R.* In conclusion, increasing energy intake could promote fetal growth and birth weight, with greater intestinal morphology and enzyme activities.

## 1. Introduction

Feeding during gestation is critical to the development and growth of the fetus and corresponding tissues. The evolutionary biology of sows means that it is predisposed to require more energy in gestation. Therefore, increased energy intake during late gestation can positively affect fetal growth and maternal weight gain. It has been concluded that additional feed in late gestation can improve the reproductive performance, the study from which showed a greater body weight (BW) and weaned weight [1]. Kongsted suggested that pregnancy rate and litter size can be influenced by energy intake [2]. It has been confirmed that the litter BW is important because of its positive correlation with pre-weaning mortality; small piglets are physiologically deprived of energy stores, and they are more susceptible to hypothermia and have a lower capacity to maintain body temperature post-birth [3,4]. The gastrointestinal tract (GIT), as an internal organ to digest nutrients and resist exogenous antigens, starts to develop at early gestation and mature rapidly in late gestation for extra-uterine life [5]. The functional maturation of GIT occurs in both pre- and postnatal period, which is largely influenced by maternal nutrition [6,7]. It has been suggested that maternal nutrition levels affect fetal intestinal development by regulating digestive enzyme activities and the gene expression of transporters in both newborn and weaned piglets [8]. Additionally, maternal energy restriction decreases the offspring’s BW, as well as visceral organs, especially the small intestine [9]. Recent studies in sows have demonstrated that maternal over-nutrition during gestation significantly increased jejunum mucosa lactase activity in newborn offspring [8]. However, it is not known whether the compensative growth is related to positive digestion or absorption function. In this study, therefore, we investigated whether providing maternal high-energy intake would affect fetus growth and intestinal development.

## 2. Materials and Methods

### 2.1. Animals and Diet

All experimental procedures were approved by the University of Sichuan Agricultural Animal Care Committee, and were followed the current laws of animal protection (Ethic Approval Code: SCAUAC201308-1). Twenty purebred Large White (LW) gilts with an average weight of 135.54 ± 0.66 kg were artificially inseminated three times with the identical semen of a purebred Large White boar at the third observation of estrus. The day of the last insemination constituted the first day of gestation. Sows were randomly assigned to one of two groups after mating to receive different isoprotein corn-wheat bran-soya bean meal-based diets: The control (CON) diet was designed as per the recommendations of the National Research Council (NRC; 2012) for gestating sows, and the high-energy diet (HED) exchanged fiber that comprised 4.6% of the total diet weight replaced with the same percentage of soy bean oil to increase energy intake by 13%. Sows were fed 2.0 kg/day during early pregnancy (days 1–30 of gestation), 2.4 kg/day mid-pregnancy (days 31–90), and 3.0 kg/day late pregnancy (days 91 to parturition). After farrowing, sows were fed a lactation diet *ad libitum* according to the NRC 2012. All sows during gestation were housed in individual feed stalls and, during lactation, were housed in farrowing pens.

### 2.2. Blood Sampling and Analyses

The BWs were measured and blood samples were collected from the pregnant gilts and fetuses (before the morning feed; *n* = 6) on the 90th day of pregnancy, at the neonate phase, and after being weaned. Blood samples were collected via acute jugular venipuncture and placed in heparinized test tubes. All blood samples were centrifuged immediately (3000× *g* for 15 min at 4 °C). Serum samples were collected and stored at −80 °C for further analysis.

### 2.3. Tissue Sample Collection

On day 90 of gestation, four gilts were weighed and slaughtered at a local abattoir after deep anesthesia with Zoletil 50 (Zoletil 50 Vet, Virbac, France) at a dose of 0.1 mg/kg BW administered by intramuscular injection. The uterus was removed from the gilts, and the fetuses were collected. The number and weight of the fetuses were measured. On the day of parturition, the number and the BW of the neonates were recorded immediately before colostrum. Each group consisted of 6 piglets that were obtained from 3 pregnant gilts. Those piglets were anesthetized with an intravenous injection of Zoletil 50 at a dose of 0.1 mg/kg BW and slaughtered after recording the weight. Of the remaining piglets, 2 average-weight piglets of each litter were selected at the point of being weaned, and the rest of the piglets were anesthetized with an intravenous injection of Zoletil 50 at a dose of 0.1 mg/kg BW and slaughtered after recording the weight. The length and weight of the small intestine were measured without luminal contents. Duodenal, jejunal, and ileal samples with a length of approximately 2 cm were cut and fixed in a 4% paraformaldehyde solution for histological analyses. The rest of the jejunum, ileum, and duodenum were snap-frozen in liquid nitrogen and stored at −80 °C for further analysis.

### 2.4. Small Intestinal Morphology

Four percent paraformaldehyde fixed duodenal, jejunal, and ileal of fetuses, newborn piglets, and weaned piglets were dehydrated and embedded in paraffin. Samples were cut into 5 μm slices on microtome. Slices were then stained with hematoxylin and eosin for intestinal morphology measurement of 20-well oriented villi and crypts (Optimus software version 6.5; Media Cybernetics, Silver Spring, MD, USA), and the villus to crypt ratios (VCR) was then calculated.

### 2.5. Enzyme Analyses

Frozen jejunum samples were weighed and homogenized in 9 times the volume of 50 mM of Tris-HCl buffer (pH 7.0) on ice with a homogenate machine (Homogenizer Power Gen 125™, Fisher Scientific, Pittsburgh, PA, USA). Homogenate was centrifuged at 3000 g and 4 °C for 10 min, and the supernatant was collected and stored at −20 °C for an enzyme assay. Total protein was extracted, and the concentration was determined according to the manufacturer’s instructions (Nanjing Jiancheng Bioengineering, Nanjing, China). Disaccharidase (including maltase, sucrase, and lactase) activities were measured with commercial kits according to the manufacturer’s instructions (Nanjing Jiancheng Bioengineering, Nanjing, China). The absorbance was determined with a spectrophotometer (Beckman Coulter DU-800; Beckman Coulter, Inc., Brea, CA, USA). The activities of disaccharidases were presented as U/mg protein. One unit (U) was defined as 1 nmol of maltose, sucrose, and lactose for the enzymatic reaction, which was instituted according to the manufacturer’s instructions [8].

### 2.6. Total RNA Extraction and Real-Time RT-PCR

Total RNA of frozen jejunum and ileum samples were extracted by using Trizol (catalogue No. 15596-026; Invitrogen, Waltham, MA, USA) according to the manufacturer’s instructions. The quality and purity of the RNA samples were assessed via electrophoresis with 1.0% agarose gel and a nucleic acid analyzer (A260/A280, Beckman DU-800; Beckman Coulter, Inc., Brea, CA, USA), respectively. Impurity of DNA in the samples was erased by DNase (Takara, Shiga, Japan). Reverse template PCR was then performed with a Prime Scripte RT reagent kit (cat# RR047A; Takara, Shiga, Japan) and 1 μg of RNA per 20-μL volume at 37 °C for 15 min, followed by 85 °C for 5 s to inactivate the enzyme. 1-μL RT-PCR products were used for real-time PCR. Real-time PCR was performed on an ABI-7900HT instrument (Applied Bio systems, Foster City, CA, USA) with the SYBR green mix system (cat#RR820A; Takara, Shiga, Japan). The sequences of the primers and the length of the products are presented in Table 1. The reaction mixture (10 μL) contained 4.8 μL of fresh SYBR^®^ *Premix Ex*Taq™II (TliRNaseH Plus, Shiga, Japan) and 0.2 μL of ROX Reference Dye II (50×), 1 μL of the primers, 1 μL of RT products, and 3 μL of diethylpyrocarbonate-treated water. The following PCR protocol was used: one cycle (95 °C 30 s); forty cycles (95 °C 5 s, 60 °C 31 s); and one cycle (95 °C 15 s, 60 °C 1 min and 95 °C 15 s). The correlation coefficients (r) of all the standard curves were >0.99, and the amplification efficiency values were between 90% and 110%. At the end of amplification, a melting curve analysis was performed to identify amplification specificity. 28S transcript was used to standardize the results by eliminating variations in mRNA and complementary DNA quantity and quality, and each mRNA level was expressed as its ratio to 28S mRNA. The relative quantification of gene expression among the treatment groups was analyzed via the 2^−ΔΔCt^ method [10].

### 2.7. Western Blot Analysis

For total protein extraction, frozen jejunum was homogenized with cell lysis buffer for Western blot analysis, and IP (cat#p0013; Beyotime, Shanghai, China) was supplemented with protease inhibitor cocktail (Roche Diagnostics Ltd., Shanghai, China). The homogenate was then centrifuged for 30 min (12,000 rpm at 4 °C). The supernatant was transferred to a new tube, and the protein concentration was measured with a BCA Protein Assay Kit (cat#p0012, Beyotime) on a plate reader. Protein lysates were boiled with sample buffer (Bio-rad, Hercules, CA, USA) and then separated on 10% SDS-PAGE gel. The separated protein was then transferred to an apolyvinylidene fluoride (PVDF) membrane. The membrane was blocked in TBS-T buffer (50 mM Tris-HCl, 150 mM NaCl, 0.1% Tween, pH 7.6) supplemented with 5% non-fat dry milk at room temperature for 1 h, followed by incubation overnight at 4 °C with the indicated primary antibody: *α-Tubulin* (DM1A) *Mouse* *mAb* (1:10,000) (cat#3873, Cell Signaling, Danvers, MA, USA), *IGF-I Receptor β* (111A9) *Rabbit* *mAb* (1:1000) (cat#3018, Cell Signaling), *IGF-IIR receptor* (H-300) (1:200) (sc-25462, Santa Cruz, CA, USA). The next morning, the membrane was washed for 5 min in TBS-T six times, and was then incubated with HRP-linked secondary antibody *Anti-mouse IgG* (1:2000) (cat#076, Cell Signaling) or *Anti-rabbit IgG* (1:2000) (cat#7074, Cell Signaling) in TBS-T for 1 h at room temperature. The signal was detected with chemiluminescent HRP substrate (Bio-rad).

### 2.8. Statistical Analysis

SPSS 21.0 (IBM SPSS Company, Chicago, IL, USA) was used for all statistical analyses. Results were tested for variance using Levene’s test, and variables that were not normally distributed were transformed (using log10 function) prior to statistical analyses. A two-tailed *t*-test was used to compare the differences between the CON and the HED. All data are shown as mean ± SEM, and differences between treatments were considered significant when *p* < 0.05.

## 3. Results

### 3.1. The BW and Small Intestine (SI) Index of Offspring Increased in the HED Group

As shown in Table 2, dams fed HED diet gained more bodyweight than that fed CON diets (+13.35 kg, *p* < 0.005). In comparison with the CON group, fetuses or piglets from the HED group had greater BW in fetuses (+20%, *p* < 0.05), birth piglets (+19%, *p* < 0.05), and weaned piglets (+25%, *p* < 0.01) and had greater BW on day 90 (+6%, *p* =0.05) and 114 (+5%, *p* < 0.05) in sows. The weight of the offspring’s small intestine (SI) increased by 60% (*p* < 0.001), 36% (*p* < 0.05), and 16% (*p* < 0.05) on day 90 of gestation, day 1 post-birth, and day 28 post-birth, respectively, in the HED group compared with the CON group. Additionally, the SI length and the ratio of SI weight to length increased 33% (*p* < 0.01) and 45% (*p* < 0.01), respectively, on day 90 of gestation in the HED group compared with the CON group, but no significant differences were observed on day 1 post-birth or day 28 post-birth. However, there was no difference between the HED group and the CON group in the ratio of SI weight to BW on day 90 of gestation, day 1 post-birth, or day 28 post-birth (*p* > 0.05) (Table 2).

### 3.2. Intestinal Morphology Improved in the Offspring of HED-Fed Mothers

**Figure 1:**
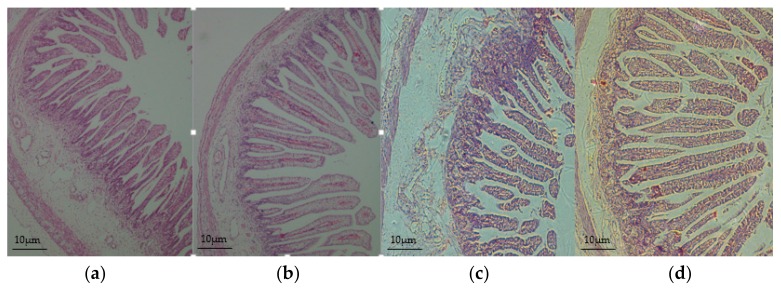
Jejunal histomorphology of fetuses and newborn piglets. (**a**,**c**) indicate the control diet (CON); (**b**,**d**) indicate the high-energy diet (HED). (**a,b**) show the fetuses’ jejunal histomorphology; (**c,d**) show the newborn piglets’ jejunal histomorphology. Intestinal villi in (**a**,**c**) were shorter compared with the same period (**b**,**d**). Original magnification: 100×. (*n* = 6 for each group).

The villus height were significantly increased at the segment of jejunum in the HED group on day 90 of gestation (+15%, *p* < 0.05) and on day of birth (+15%, *p* < 0.01) than the CON group (Figure 1 and Table 3). As for ileum had significantly increased villus height in HED only at day 90 of gestation fetuses (+18%, *p* < 0.05) and weaned piglets (+12%, *p* < 0.05). Villus height, crypt depth, and VCR of the duodenum segments showed no significant difference at the three time points between the HED group and the CON group.

### 3.3. The Activities of Digestive Enzyme Increased in the Jejunum and Ileum of the Offspring of HED-Fed Mothers

The activity of lactase in jejunum markedly increased (+68%, *p* < 0.05) in the HED group on day 28 post-birth than the CON group, while there was no difference on day 1 post-birth or day 28 post-birth (Table 4). As a complement, the activity of lactase in ileum markedly increased (+50%, *p* < 0.05) in the HED group on day 1 post-birth, while there was no difference on day 28 post-birth, as compared with the CON group. The activity of sucrase in the HED group markedly increased on the days 1 and 28 post-birth both in jejunum and ileum more than the CON group (*p* < 0.01) (Table 4). The activity of maltase showed no difference between the two groups in jejunum and ileum at any of the three time points.

### 3.4. Serum Insulin-Like Growth Factor 1 (IGF-1) Concentration Increased in the Offspring of HED-Fed Mothers

**Figure 2:**
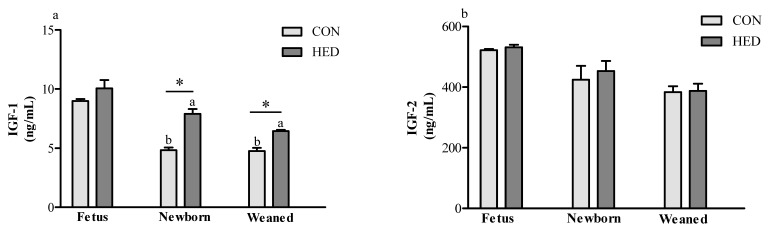
The expression of insulin growth factor 1 (IGF-1) and insulin growth factor 2 (IGF-2) in the serum of fetuses, newborn piglets, and weaned piglets born to sows fed different energy levels during gestation. (**a**) indicates IGF-1 concentration in sera; (**b**) indicates IGF-2 concentration in sera. (*n* = 6 for each group). * indicates *p* < 0.05 HED group *vs.* CON group.

As shown in Figure 2, compared to the CON group, the IGF-1 concentration in newborn and weaned piglets were significantly increased by 63% (*p* < 0.05) and 36% (*p* < 0.05), respectively, in the HED group. However, no significant effects of a maternal high-fat diet intake during gestation was observed in the offspring’s insulin-like growth factor 2 (IGF-2) concentration on day 90 of fetus, day 1 post-birth, or day 28 post-birth between the HED group and the CON group.

### 3.5. The Expression of Growth Factors Increased in Jejunum

**Figure 3:**
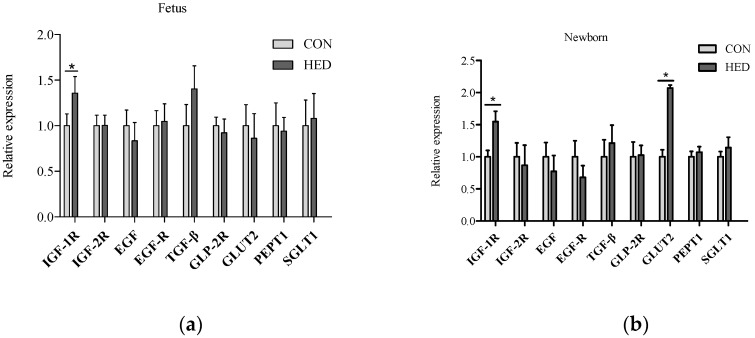

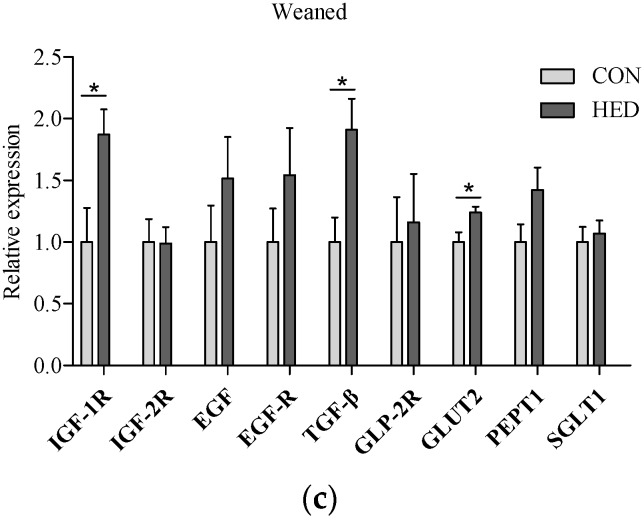
The mRNA expression of growth factor and nutrient transporters in the jejunum of fetuses (**a**), newborn piglets (**b**); and weaned piglets (**c**) born to sows fed different energy levels during gestation. *IGF-1*: insulin-like growth factor 1; *IGF-2*: insulin-like growth factor 2; *IGF-1R*: insulin-like growth factor 1 receptor; *IGF-2R*: insulin-like growth factor 2 receptor; *EGF*: epidermal growth factor; *EGFR*: epidermal growth factor receptor; *TGF-β*: transforming growth factor-β; *GLP-2R*: glucagon-like peptide-2 receptor; *SGLT1*: Na^+^-dependent glucose transporter 1; *GLUT2*: glucose transporter 2; *PEPT1*: peptide transporter 1. (*n* = 6 per group). * indicates *p* < 0.05 HED group *vs.* CON group.

**Figure 4:**
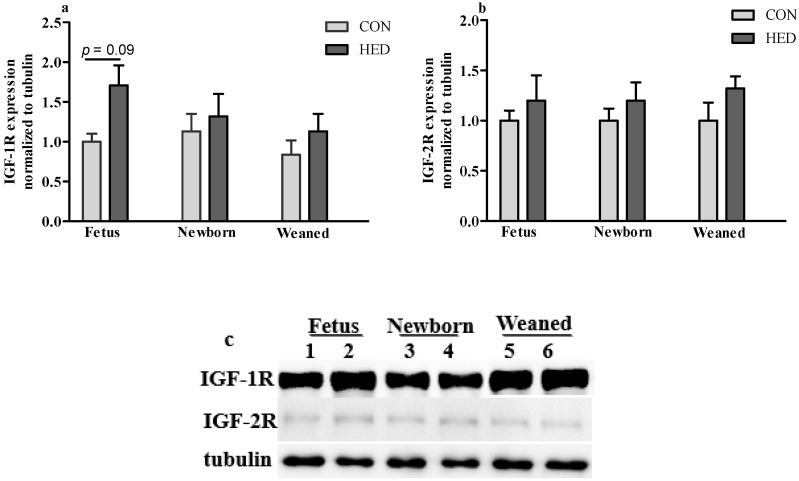
Protein expression of IGF-1R and IGF-2R. (**a**) Relative protein expression of IGF-1R and (**b**) IGF-2R in the jejunum of fetuses, newborn piglets, and weaned piglets. (*n* = 4 per group); (**c**) Selected western blot for IGF-1R, IGF-2R and tubulin. 1, 3, 5 for CON, 2, 4, 6 for HED. IGF-1/2R, insulin-like growth factor 1/2 receptor.

The gene expression levels of *IGF-1R* significantly increased in the jejunum in the maternal HED intake group in fetuses on day 90 of gestation, the newborn piglets, and the weaned piglets (*p* < 0.05) (Figure 3). Additionally, the *TGF-β* mRNA expression level increased in the jejunum of weaned piglets in the HED group (*p* < 0.05). Moreover, the glucose transporter *GLUT2* mRNA expression levels significantly increased in the jejunum of the newborn piglets and the weaned piglets in the HED group (*p* < 0.05). The protein expression levels of *IGF-1R* and *IGF-2R* were higher in the jejunum in the fetuses, the newborn piglets, and the weaned piglets of the HED group than in that of the CON group, but the difference was not significant between the two groups (Figure 4).

## 4. Discussion

In recent years, the “Barker Hypothesis” or the “developmental origins of adult disease” concept, which addresses that risk patterns for both obesity and type 2 diabetes originate as a consequence of alterations in growth and metabolism during critical windows of intrauterine life, has become well established [11,12]. In particular, maternal obesity or high-fat diet can propagate the risk of metabolic syndrome to subsequent generations via nongenetic or epigenetic and metabolic mechanisms [12]. In those studies, the role of specific tissue or organs, e.g., liver, skeletal muscle and pancreas, has been examined, the role of intestinal adaptation to maternal over-nutrition in the control of developmental risk of metabolic syndromes, however, was not clearly. In nutritional programming studies, rodents are the most frequently used model for man, but the developmental pattern of porcine gastrointestinal tract is much closer to the human than that of rodents. In the present study, the dams fed HED diet not only gained greater bodyweight, but also resulted in a higher circulating triglyceride concentration (0.45 ± 0.04 mmol/L and 0.54 ± 0.02 mmol/L in CON and HED diets, respectively, *p* = 0.065) compared with CON dams during pregnancy, suggesting metabolic dysfunction state of dams fed HED.

Fetuses receive constant nutrients from mothers via the placenta, whereas pups must uptake nutrients from food via the SI post-birth; therefore, the SI development during the gestation period plays an important role. Maternal food intake not only helps to maintain the pregnancy but also affects the growth of fetuses. Studies have confirmed the direct relationship between maternal nutrition and fetal weight [13,14]. Extra food or energy during late gestation can marginally improve BW. A study on female has shown that, during prenatal and early postnatal life, the nutritional state of the mother can induce long-term metabolic changes and increase susceptibility to metabolic disease in later life, which also has effects on fetal/offspring weight and development in later life [15]. In this study, maternal HED intake during gestation significantly increased sow and piglet BW and SI indexes, such as SI weight, SI length, and the SI weight to length ratio. A similar result was also reported by Cao *et al.*, who demonstrated that the higher level of maternal food intake induced a higher SI weight, ratio of weight to length, and SI villus height [8]. The higher BW caused by maternal over-nutrition generally lasts through postnatal life. It has been reported that neonates with a higher BW are vulnerable to a greater future BW, which may suggest an increase in digestive and absorptive capabilities [16]. Accordingly, our data shows that maternal HED intake during gestation improved the intestinal morphology and digestive enzyme activity in the fetal or neonatal piglets. In terms of digestive capacity, intestinal enzymes are partially responsible for food processing and hydrolyzing macromolecule nutrients to small molecules for intestinal absorption [17]. During intrauterine life, glucose and amino acids were the main nutrients to provide energy for intrauterine growth, however, the piglets utilize abundant lactose in milk to provide most of the energy during the neonatal life [18,19]. In this study, the activities of lactase and sucrase were notably increased in the small intestine of weaned piglets in the HED group, indicating that a heavier offspring from HED dams might have adaptively programed a higher digestive activity for more energy to meet the needs of neonatal growth. A reduction in the ratio of jejunal villus height to crypt depth indicates that the intestinal digestion and absorption function are affected. The recent studies on IUGR piglets provided further evidence for this [20]. However, although maternal high-fat diet intake could cause the jejunal villus to be higher at birth, in this study, maternal nutrition intake did not markedly affect the ratio of jejuna villus height to crypt depth of the offspring’s SI at the point of being weaned. Meyer *et al.* showed a similar result that demonstrated that maternal nutritional levels during gestation had no significant effect on jejunal crypt depth and the villus length of neonatal lamb [21]. Based on the information, it may be concluded that the ratio of jejuna villus height to the crypt depth of the SI could not be significantly affected by a maternal high-fat diet during gestation.

Growth factors such as *IGF-1* and its receptor are important molecules to promote intestinal development and its function formation. During late pregnancy in sows, it is a vital period of mucosal maturation and cell differentiation in the SI [19]. Additionally, amniotic fluid and colostrum contain several growth factors, including insulin-like growth factor-1 (*IGF-I*), epidermal growth factor (*EGF*), and transforming growth factor-β (*TGF-β*). The concentrations are changed, which may provoke small intestinal growth [22]. In this study, we demonstrated that serum IGF-1 concentration significantly increased in newborn and weaned piglets, and in the mothers. Maternal nutrient intake is a dominant influence on IGF-1 concentrations prenatally, and the correlation between BW and IGF-1 is quite similar [23]. Furthermore, the mRNA expression level of *IGF-1R* in the jejunum of 90-day-old fetuses and neonates were significantly higher in the HED group. This was consistent with the previous studies, which suggest that IGF-1 concentration and *IGF-1R* mRNA abundance may enhance fetal growth and gut development and improve SI cell differentiation [24]. *TGF-β* mRNA also increased in the jejunum of fetuses, newborn piglets, and weaned piglets by the maternal HED intake. The protein expression levels of *IGF-1R* in jejunum tended to be higher in the HED group offspring. Based on this information, it can be concluded that the growth factor expressions in the SI may significantly regulate SI cell proliferation and differentiation, which is affected by maternal HED intake during gestation.

Consistently, the increasing intestinal absorption rates in obese animals are related to an overexpression of nutrient transporters [25]. In the present study, it should also be noted that the glucose transporter protein abundance in the whole tissue of an offspring’s jejunum samples tended to increase with the HED-fed group. To our knowledge, glucose is a major substrate to provide energy for fetal growth and development and can be absorbed from the diet in the proximal jejunum by 2 different routes: the *GLUT2* and the *SGLT1* [26]. In this study, a maternal HED significantly increased intestinal gene expression of *GLUT2* in newborn and weaned piglets, which is consistent with Gabler [27]. Moreover, the expression of *PEPT1* increased in piglets in the HED group during the entire life cycle. The mRNA expression of *GLUT2* was upregulated, which may enable the acquisition of more energy for maintenance requirement. The effect of the maternal HED on the mRNA expression of transporters lasted until being weaned. The present results indicate that these animals would grow faster after being weaned by enhanced intestinal absorption.

## 5. Conclusions

Our study demonstrates that increasing energy intake by adding soybean oil during gestation does have significant consequences on an offspring’s intrauterine growth, especially the development of its small intestine, as well as its digestive capacity, which was sustained post-birth. Moreover, this process is mainly mediated by growth factor *IGF-1R*. Based on the results revealed by the present study, the intestine could be considered as a possible target to modulate the development of metabolic dysfunction in the offspring born from mothers with over-nutrition during pregnancy.

## Figures and Tables

**Table 1:** Oligonucleotide primers used for a relative-Quantitative real-time PCR analysis.

Primers	Sequences (5′-3′)		GenBank Accession
28S	Sense	TACCCATATCCGCAGCAGGTC	4W24_5
	Antisense	CCCTTAGAGCCAATCCTTATCCC	
*IGF-1R*	Sense	GGAGGAAGTGACAGGGACTAAAGG	NM_214172.1
	Antisense	GGTGCCAGGTGATGATGATGC	
*IGF-2R*	Sense	CGCTTTCATCATCCGCTTCG	JQ250827.1
	Antisense	CAGCGCGGTTTCAAAGTCAA	
*EGF*	Sense	TCCTGTCAGCTAACCCATTACG	NM_214020.1
	Antisense	TGAGTTACCGAGTGATTCTCCC	
*EGFR*	Sense	GGGATAGGGATTGGCGAGTT	NM-2140075
	Antisense	GCCACGTATGATTTCCAGGTTC	
*TGF-β*	Sense	AAGCGGCAACAAAATCTATG	AF101033.1
	Antisense	CCCGAGAGAGCAATACAGGT	
*GLP-2-R*	Sense	TGGCAGGACGACTCCCAGTG	NM_001246266.1
	Antisense	CAGGATGAAAGAGGCAAACAGG	
*SGLT1*	Sense	CCACTTTCCCTATAAAACCTCAC	NM_001164021.1
	Antisense	CTCCATCAAACTTCCATCCTCAG	
*GLUT2*	Sense	CCTGCTTGGTCTATCTGCTGTG	NM_001097417.1
	Antisense	TTGATGCTTCTTCCCTTTCTTT	
*PEPT1*	Sense	GATGAAATGTGAGCGTATGGG	AY180903.1
	Antisense	AAAGAGGGAGGATCTGGAAAA	

PCR: polymerase chain reaction; *IGF-1R*: insulin-like growth factor 1 receptor; *IGF-2R*: insulin-like growth factor 2 receptor; *EGF*: epidermal growth factor; *EGFR*: epidermal growth factor receptor; *TGF-β*: transforming growth factor-β; *GLP-2R*: glucagon-like peptide-2 receptor; *SGLT1*: Na^+^-dependent glucose transporter 1; *GLUT2*: glucose transporter 2; *PEPT1*: peptide transporter 1.

**Table 2:** The effect of sows fed different energy levels during pregnancy on the growth performance of offspring at day 90 of gestation, day 1 post-birth, and day 28 post-birth. (Mean values with their standard errors).

Parameter	Fetus			Newborn Piglets			Weaned Piglets		
	CON	HED	*p-*Value	CON	HED	*p-*Value	CON	HED	*p-*Value
BW (Kg)	0.65 ± 0.03	0.78 ± 0.01	0.016	1.33 ± 0.21	1.58 ± 0.13	0.031	5.53 ± 0.31	6.91 ± 0.29	0.009
SI (cm)	96.23 ± 3.65	110.45 ± 4.60	0.053	277.17 ± 50.63	288.67 ± 14.51	0.604	653.38 ± 38.77	738.67 ± 35.30	0.135
SI (g)	9.93 ± 0.80	15.84 ± 1.44	<0.001	34.87 ± 6.72	47.65 ± 7.80	0.013	200.30 ± 6.23	233.43 ± 12.56	0.040
SI (cm·kg^−1^ BW)	151.48 ± 4.85	144.89 ± 6.13	0.446	210.83 ± 18.70	183.74 ± 5.56	0.195	119.06 ± 6.23	107.57 ± 5.65	0.202
SI (g·kg^−1^ BW)	15.63 ± 0.50	20.80 ± 1.00	0.004	26.10 ± 1.13	30.20 ± 1.68	0.071	36.91 ± 2.69	33.94 ± 1.80	0.380
SI weight/length (mg·cm^−1^)	103.19 ± 4.25	137.41 ± 3.47	0.002	128.36 ± 12.40	164.73 ± 9.50	0.042	311.47 ± 19.01	315.66 ± 4.37	0.834
**Parameter**	**D 0**			**D 90**			**D 114**		
	**CON**	**HED**	***p-*Value**	**CON**	**HED**	***p-*value**	**CON**	**HED**	***p-*Value**
Sows BW (Kg)	135.60 ± 0.80	135.48 ± 1.07	0.927	189.30 ± 1.92	202.65 ± 3.55	0.005	210.85 ± 6.19	221.93 ± 13.01	0.049

BW: body weight; SI: small intestine. (*n* = 6 for each group).

**Table 3:** Intestinal development and morphometric measurements (μm, in duodenum, jejunum, and ileum) in offspring of sows fed different energy levels during pregnancy, as a neonate, and after being weaned (28 day post-birth). (Mean values with their standard errors). (*n* = 6 for each group).

Parameter	Fetus			Newborn Piglets			Weaned Piglets		
	CON	HED	*p-*Value	CON	HED	*p-*Value	CON	HED	*p-*Value
Jejunum									
Villus height (μm)	25.62 ± 1.12	29.45 ± 1.43	0.033	109.65 ± 4.77	126.06 ± 3.24	0.004	68.58 ± 7.69	72.66 ± 3.36	0.182
Crypt depth (μm)	3.82 ± 0.26	4.45 ± 0.38	0.791	22.37 ± 0.51	23.39 ± 0.55	0.212	33.73 ± 0.90	38.87 ± 1.09	<0.001
VCR	5.90 ± 0.36	6.98 ± 0.66	0.174	2.08 ± 0.28	5.17 ± 0.25	0.001	2.15 ± 0.07	2.18 ± 0.09	0.787
Ileum									
Villus height (μm)	24.12 ± 1.20	28.44 ± 1.23	0.043	112.05 ± 4.10	113.22 ± 4.82	0.854	54.02 ± 1.54	60.28 ± 2.31	0.031
Crypt depth (μm)	3.92 ± 0.26	4.05 ± 0.38	0.791	33.48 ± 1.82	34.11 ± 1.84	0.606	27.40 ± 0.86	30.17 ± 1.50	0.098
VCR	6.90 ± 0.36	7.98 ± 0.66	0.174	3.75 ± 0.16	4.12 ± 0.25	0.189	2.03 ± 0.14	2.45 ± 0.09	0.014
Duodenum									
Villus height (μm)	15.62 ± 1.12	19.45 ± 1.43	0.333	76.55 ± 1.91	84.31 ± 4.48	0.066	105.21 ± 2.70	103.87 ± 3.36	0.757
Crypt depth (μm)	4.42 ± 0.26	4.45 ± 0.38	0.791	38.42 ± 0.84	38.37 ± 1.06	0.970	54.56 ± 1.33	50.54 ± 1.54	0.054
VCR	2.90 ± 0.36	2.98 ± 0.66	0.874	2.08 ± 0.07	2.26 ± 0.12	0.148	2.10 ± 0.08	2.22 ± 0.10	0.367

**Table 4:** Effect of different energy level during gestation on activity of different digestive enzymes in small intestine (in jejunum and ileum) of piglet fetuses, and newborn and weaned piglets (28 day post-birth). (Mean values with their standard errors). (*n* = 6 for each group).

U/mg Protein	Fetus			Newborn Piglets			Weaned Piglets		
	CON	HED	*p-*Value	CON	HED	*p-*Value	CON	HED	*p-*Value
Jejunum									
Lactase	105.91 ± 10.21	68.71 ± 2.36	0.012	96.89 ± 2.52	93.57 ± 44.38	0.943	50.61 ± 6.34	85.08 ± 11.86	0.043
Maltase	35.73 ± 3.71	32.14 ± 2.15	0.435	23.59 ± 1.49	30.47 ± 3.83	0.145	92.93 ± 46.47	105.16 ± 52.56	0.115
Sucrase	-	-	-	1.42 ± 0.17	4.37 ± 0.18	<0.001	66.88 ± 5.74	151.12 ± 18.33	0.005
Ileum									
Lactase	33.40 ± 4.73	28.49 ± 4.56	0.483	10.83 ± 0.99	20.48 ± 0.91	<0.001	0.12 ± 0.02	0.18 ± 0.03	0.199
Maltase	16.84 ± 1.78	16.85 ± 0.81	0.996	13.82 ± 1.43	16.46 ± 2.29	0.366	50.32 ± 10.78	79.87 ± 11.02	0.104
Sucrase	-	-	-	0.22 ± 0.04	1.35 ± 0.21	0.002	8.67 ± 0.76	17.81 ± 1.19	0.001
